# Investigation of human haemotropic *Mycoplasma* infections using a novel generic haemoplasma qPCR assay on blood samples and blood smears

**DOI:** 10.1099/jmm.0.021691-0

**Published:** 2010-11

**Authors:** Séverine Tasker, Iain R. Peters, Andrew D. Mumford, Michael J. Day, Timothy J. Gruffydd-Jones, Sarinder Day, Anne-Marie Pretorius, Richard J. Birtles, Chris R. Helps, Harold Neimark

**Affiliations:** 1 School of Veterinary Sciences, University of Bristol, Langford, Bristol BS40 5DU, UK; 2 Bristol Heart Institute, Bristol Royal Infirmary, Bristol BS2 8HW, UK; 3 University of the Free State, Bloemfontein, South Africa; 4 University of Liverpool, Chester High Road, Chester CH64 7TE, UK; 5 State University of New York, Brooklyn, NY, USA

## Abstract

The aim of this study was to develop quantitative real-time (q)PCR assays to detect all known haemoplasma species, and a human housekeeping gene in order to demonstrate both successful DNA extraction from clinical samples and to test for sample inhibition, and to apply these qPCRs to human blood samples and blood smears. Sensitive and specific generic haemoplasma qPCR assays were developed to amplify haemoplasma species, as well as human glyceraldehyde-3-phosphate dehydrogenase (GAPDH) as an internal amplification control. An optimized technique for extracting DNA from stained blood smears was also developed. These methods were applied to anonymized blood samples obtained from 100 human immunodeficiency virus (HIV)-infected South Africans and 920 UK patients undergoing haematological examination, and to 15 blood smears recruited from previous studies describing human haemoplasmosis. Human GAPDH levels were acceptable in all but three of the blood samples and all but two of the blood smears. The latter could have arisen due to DNA degradation due to the old age (over 35 years) of these smears. Haemoplasma infection was found in one HIV-infected South African, but the species could not be characterized due to the very low levels of DNA present. This report describes novel extraction and qPCR methodologies for haemoplasma screening. Previously reported human haemoplasmosis based on cytological diagnosis alone should be viewed with caution.

## INTRODUCTION

Haemotropic mycoplasmas (‘haemoplasmas’) are wall-less erythrocytic bacteria that have not yet been cultured *in vitro*. Haemoplasmas are classified within the genus *Mycoplasma* based on 16S rRNA gene and RNase P RNA gene phylogeny (Neimark *et al.*, 2001, 2002; Peters *et al.*, 2008b). They are divided, based on phylogeny (rather than pathogenicity or host specificity), into two groups: a haemominutum group and a haemofelis group (Peters *et al.*, 2008b; Tasker *et al.*, 2003c). Haemoplasmas have been identified in many domestic and wild animal species and can cause haemolytic anaemia (Barker *et al.*, 2010; Hoelzle, 2008; Hofmann-Lehmann *et al.*, 2004; Neimark *et al.*, 2002, 2004; Stoffregen *et al.*, 2006; Willi *et al.*, 2007).

Diagnosis of haemoplasmosis in animals has historically relied on cytology, but the observation of organisms on erythrocytes in blood smears is known to be unreliable compared to PCR. In particular, sensitivity is very poor ranging from 0 to 37.5 % (Bauer *et al.*, 2008; Tasker, 2010; Tasker *et al.*, 2003b; Westfall *et al.*, 2001). Although specificity is higher, with values of 84–98 % reported, it must be noted that these figures are based upon experienced board-certified veterinary clinical pathologists interpreting blood smears and that lower specificity is common when smears are examined by individuals lacking experience in haemoplasma diagnosis (Bauer *et al.*, 2008; Tasker, 2010; Tasker *et al.*, 2003b). PCR is now the preferred method of diagnosis (Peters *et al.*, 2008a).

Human haemoplasma-like infections have been reported, although descriptions of diagnostic methods used and correlation with clinical disease have been poor. Human haemoplasmosis has been reported in association with systemic lupus erythematosus in the USA (Kallick *et al.*, 1972) and Croatia (Bosnic *et al.*, 2010), in AIDS patients in Brazil (Duarte *et al.*, 1992), in sick hospitalized patients in Niger, Africa (Grétillat & Konarzewski, 1978), in humans from Inner Mongolia (Yang *et al.*, 2000), in a patient with neoplasia in the UK (Clark, 1975) and in a patient with infectious mononucleosis in Yugoslavia (Puntaric *et al.*, 1986), but in all cases diagnosis was based primarily on cytology. Other cytological reports of human haemotropic infections (Archer *et al.*, 1979; Dooley, 1980; Kallick *et al.*, 1980; Puntaric *et al.*, 1994) are thought unlikely to reflect haemoplasmosis due to a lack of mycoplasmal morphological features in the bacteria.

Recently, PCR has been used to detect human haemoplasma infection. Although two studies did not give full descriptions of the origins of the samples analysed or PCR methods used (Hu *et al.*, 2009; Yang *et al.*, 2007), more thorough descriptions have been made by Yuan *et al.* (2007, 2009), where PCR detected the presence of an organism very similar to the porcine haemoplasma *Mycoplasma suis* in people in China, although clinical haemoplasmosis was not common. In another study, PCR detected *Mycoplasma haemofelis*-like DNA in the blood of a Brazilian with human immunodeficiency virus (HIV) (dos Santos *et al.*, 2008), although the clinical significance of infection was not discussed. These reports imply the existence of zoonotic haemoplasma infections.

PCR studies describing human haemoplasmosis have used assays designed to amplify a single animal haemoplasma species but it is possible that people can be infected with as-yet-unidentified haemoplasma species. One group has applied a ‘universal’ haemoplasma PCR assay to 414 human blood samples, but no positive results were obtained and the lack of an internal amplification control hindered interpretation of negative results (Willi *et al.*, 2009).

The aim of this study was to develop quantitative real-time (q)PCR assays to detect all known haemoplasma species, and a human housekeeping gene in order to demonstrate both successful DNA extraction from clinical samples and to test for sample inhibition. These assays were applied to DNA isolated from 15 blood smears recruited from studies previously documenting suspected human haemoplasma cytology (Clark, 1975; Grétillat & Konarzewski, 1978; Yang *et al.*, 2000), and blood samples from over 1000 humans in the UK and South Africa.

## METHODS

### Primer and probe design.

Nearly full-length 16S rRNA gene sequences (equivalent to bases 81–1370 of *M. haemofelis* sequence accession number U95297) for the haemoplasma species were downloaded from GenBank (http://www.ncbi.nlm.nih.gov/genbank/) and aligned using DS Gene 1.5 (Accelrys). From the alignment a consensus sequence was created for each of the two haemoplasma groups (Peters *et al.*, 2008b; Tasker *et al.*, 2003c) and used to design primers and probes for two generic haemoplasma qPCRs: one for the haemofelis group and one for the haemominutum group (Primer3Plus; http://www.bioinformatics.nl/cgi-bin/primer3plus/primer3plus.cgi) (Untergasser *et al.*, 2007). Each of the two haemoplasma group consensus sequences was then aligned against other *Mycoplasma* species which were closely related by 16S rRNA gene phylogeny [*Mycoplasma fastidiosum* (AF125878), *Mycoplasma penetrans* (BA000026), *Mycoplasma iowae* (U29676), *Mycoplasma pirum* (M23940), *Mycoplasma gallisepticum* (AE015450), *Mycoplasma pneumoniae* (U00089) and *Mycoplasma genitalium* (L43967)] (Peters *et al.*, 2008b) and assays with primer and probe sequences which had base mismatches (Table 1) against these non-target species were selected for use.

To confirm the presence of amplifiable DNA in the extracted human samples, an assay was designed to detect the human glyceraldehyde-3-phosphate dehydrogenase (GAPDH) gene (NM_002046) using Primer3Plus and the same design criteria described above.

### Blood samples and DNA extraction.

Aliquots of surplus EDTA blood from 920 samples submitted to the Haematology service at the Bristol Royal Infirmary, Bristol, UK, and from 100 South African women infected with HIV [some of whom had been previously tested for other infectious agents (Pretorius *et al.*, 2004)] were obtained. All samples were anonymized prior to analysis and were approved by the local research ethics committees (National Health Service National Research Ethics Service and University of the Free State).

DNA was extracted from 100 μl EDTA blood using either the DNeasy Blood kit (Qiagen) or the Macherey-Nagel NucleoSpin Blood kit (ABgene) as per the manufacturer's protocol. The DNA was eluted with 100 μl elution buffer. Positive controls of mixed cat blood containing the three feline haemoplasma species (*M. haemofelis*, ‘*Candidatus* Mycoplasma haemominutum’ and ‘*Candidatus* Mycoplasma turicensis’) and a human DNA sample obtained from a mouth swab, and a negative control blood sample obtained from a specific-pathogen-free cat, were included in each DNA extraction run.

### Blood smears and DNA extraction.

Samples were recruited from three previously reported studies (Clark, 1975; Grétillat & Konarzewski, 1978; Yang *et al.*, 2000) that had documented human haemoplasma infection based on cytological examination of blood smears. Whole blood samples were no longer available but preserved (methanol-fixed) blood smears were kindly donated by colleagues (Drs Clark and Yun and Dr Grétillat's family). Four smears stained with Wright–Giemsa were obtained from the study describing haemoplasmosis in humans from Inner Mongolia, China (Yang *et al.*, 2000), eight smears (one stained with Jenner–Giemsa and seven unstained) were obtained from the patient with malignant melanoma and suspected haemoplasmosis described in the Clark (1975) study and three smears stained with May Grünwald–Giemsa were obtained from patients in Niger, Africa (Grétillat & Konarzewski, 1978).

Feline blood smears that had been made in previous studies from haemoplasma-infected (*n*=72) and non-infected (*n*=12) blood of known haemoplasma copy number ml^−1^ (calculated by qPCR) (Tasker *et al.*, 2006a, b) were available for optimization of the DNA extraction technique and evaluation of any effect of staining. Thus haemoplasma copy number values (ml blood)^−1^ were available for all of the feline haemoplasma-infected blood samples that had been used to make the blood smears. Evaluation of any effect of staining on DNA extraction and subsequent PCR was investigated by staining several feline blood smears with Wright–Giemsa, Jenner–Giemsa, Giemsa or May Grünwald–Giemsa stains (six of each) and comparing results to those obtained from unstained blood smears.

A number of methods were tried (20 smears per method) to remove the blood from the slides for DNA extraction: use of a scalpel (Aubouy & Carme, 2004) or slide scraper (Sykes *et al.*, 2008), as previously described, and use of a sterile swab moistened in PBS firmly rubbed over the entirety of the slide. Use of the scalpel or slide scraper made it difficult to remove all of the blood from the slide and concern regarding laboratory contamination arose due to blood smear material becoming airborne during removal, so an optimized method using a swab moistened in PBS was used for further studies. Following removal of material from the slide, the swab was placed into a 2 ml tube containing 200 μl PBS, 200 μl Buffer BQ1 (ABgene) and 25 μl proteinase K (0.56 mg per reaction). The tube was then incubated in a shaker (Vortemp 56; Labnet) at 70 °C and 1000 r.p.m. for 15 min (overnight incubation did not improve DNA yield; data not shown). Ethanol (100 %; 210–250 μl) was then added to the tube, mixed and the solution was transferred to a spin column. Thereafter, the manufacturer's instructions (Nucleospin Blood kit) were followed, and the DNA was eluted with 100 μl elution buffer. The DNA was stored at −20 °C until use. Positive control blood smears, obtained from cats infected with either *M. haemofelis* or ‘*Candidatus* M. haemominutum’, and negative control blood smears, obtained from specific-pathogen-free cats, were included in each DNA extraction run.

### qPCR.

The generic haemoplasma and human GAPDH qPCRs were performed using Qiagen HotStarTaq Master Mix with 200 nM of each primer, 100 nM of each probe or SYBR Green 1 (1 : 100 000 final dilution; used for validation of the primers) (Sigma-Aldrich), 4.5 mM MgCl_2_ and 5 μl DNA in a total volume of 25 μl. The extraction control samples described above, as well as positive control samples of feline haemoplasma DNA of known copy number and a negative control (water), were included in each PCR run applied to DNA from blood samples and blood smears.

All qPCRs were performed in an iCycler iQ (Bio-Rad Laboratories) with an initial incubation of 95 °C for 15 min and then 45 cycles of 95 °C for 10 s and 60 °C (haemofelis and haemominutum group qPCRs) or 57.5 °C (GAPDH qPCR) for 30 s during which the fluorescence data were collected. The thermocycling protocol was extended when SYBR Green I was used by heating the samples from 75 °C to 95 °C in 0.5 °C increments with a dwell time at each temperature of 15 s during which fluorescent data were collected to create a melting curve. The melting temperatures of the products were determined with the iCycler iQ Optical System Software (v3.1; Bio-Rad Laboratories). During the optimization of the assays, reaction products from the sequence-specific plasmids and feline blood samples infected with the three feline haemoplasma species (Peters *et al.*, 2008a), as well as the mouth swab, were separated by 2 % agarose gel electrophoresis, using ethidium bromide stain, with 5 μl EasyLadder I (Bioline) and the GelDoc-It Imaging System (UVP) used to confirm that a single amplicon of the correct size (114 bp for the haemofelis group qPCR and 139 bp for the haemominutum group qPCR) was produced. Previously sequenced plasmids containing the 16S rRNA gene from *M. haemofelis* and ‘*Candidatus* M. haemominutum’ (Tasker *et al.*, 2003a) were used to test the efficiency and sensitivity of the qPCRs. A 10-fold serial dilution of each plasmid (range: 1–10^7^ copies per reaction) in triplicate was used for each of the two generic haemoplasma group-specific assays. Specificity of the assays was assessed using DNA from haemoplasma species (*M. haemofelis*, *Mycoplasma haemocanis*, ‘*Candidatus* M. turicensis’, *Mycoplasma coccoides*, ‘*Candidatus* M. haemominutum’, ‘*Candidatus* Mycoplasma haematoparvum’, *M. suis*, ‘*Candidatus* Mycoplasma haemolamae’, *Mycoplasma wenyonii* and *Mycoplasma haemomuris*) either obtained from a previous study (Peters *et al.*, 2008b) or kindly donated by Drs Rikihisa and Messick, and non-target *Mycoplasma* species (*M. fastidiosum*, *M. penetrans*, *M. iowae*, *M. pirum*, *M. gallisepticum*, *M. pneumoniae* and *M. genitalium*) kindly provided from the *Mycoplasma* culture collection at the Veterinary Laboratories Agency (Weybridge, Surrey, UK).

A dilution of human genomic DNA obtained from a mouth swab (10-fold dilutions over 6 orders of magnitude) was used to assess the reaction efficiency of the human GAPDH qPCR.

For evaluation and optimization of the blood smear extraction method, DNA extracted from the feline blood smears was subjected to each of the two new haemoplasma haemofelis and haemominutum group-specific qPCRs described above, as well as to the previously described qPCR assays duplexed for either *M. haemofelis* or ‘*Candidatus* M. haemominutum’ and feline 28S rDNA (Peters *et al.*, 2008a) using 5 μl extracted DNA per PCR. This enabled comparison of different extraction methods by comparing haemoplasma and 28S rDNA via threshold cycle (*C*_t_) values. These results obtained on blood smears were also compared with the known contemporaneous haemoplasma copy numbers in the feline blood used to make the smears, taking into account the volume of blood used to make a blood smear (approx. 5 μl), to allow a measure of DNA recovery from the blood smear to be made. Any qPCR inhibitory effects of staining were also assessed by incorporation (‘spiking’) of a defined amount of a synthetically constructed reference plasmid, based on feline herpes virus, into the PCR mastermix, adding 5 μl of the extracted DNA from stained smears or water, and subjecting to a qPCR specific for the reference plasmid (data not shown; further information may be available from the corresponding author). The *C*_t_ values obtained using water were then compared to those obtained in the presence of DNA extracted from stained smears to determine the presence of PCR inhibition.

Acceptable human GAPDH qPCR *C*_t_ values were defined as those ≤30 on the blood samples and those ≤33 on blood smears, based on previous work with internal amplification controls (Tasker *et al.*, 2009).

## RESULTS

### Generic haemoplasma assay optimization

The generic haemoplasma haemofelis and haemominutum group-specific qPCRs primer and probe sequences are shown in Tables 1 and 2.

The assays, when run with SYBR Green I and DNA from feline blood infected with each of the feline haemoplasma species, produced a single melt peak and a single amplicon of expected size (114 bp for the haemofelis group qPCR and 139 bp for the haemominutum group qPCR) when separated by agarose gel electrophoresis (data not shown). The reaction efficiencies of the haemofelis group and haemominutum group assays were both in excess of 97 % (Table 2) and were able to detect between 1 (1/3 replicates) and 10 (3/3 replicates) plasmid copies per PCR. Of the haemoplasma species tested, the haemominutum group assay only detected ‘*Candidatus* M. haemominutum’, ‘*Candidatus* M. haematoparvum’, *M. suis*, ‘*Candidatus* M. haemolamae’ and *M. wenyonii*. The haemofelis group assay was able to detect *M. haemofelis*, ‘*Candidatus* M. turicensis’, *M. haemocanis*, *M. coccoides* and *M. haemomuris*, all members of the haemofelis group. However, ‘*Candidatus* M. haemominutum’ and ‘*Candidatus* M. haematoparvum’, both members of the haemominutum group, were also amplified by this qPCR, but much less efficiently (*C*_t_ values were approximately three to four cycles later than those obtained with the haemominutum group assay). No cross-reactivity of either the haemofelis group or the haemominutum group qPCR with DNA from the non-target *Mycoplasma* species tested was observed.

The efficiency of the human GAPDH qPCR assay was over 99 % over 6 orders of magnitude (Table 2).

### Blood samples

One of the DNA extracts from the 920 UK human blood samples was negative with the human GAPDH qPCR whilst the remaining samples had a median *C*_t_ of 22 (range 19.3–32.2). Two samples had *C*_t_ values >30 (31.9 and 32.2). Thus GAPDH levels were acceptable in all but three of the 920 blood samples. None of the samples were positive with either of the two generic haemoplasma group qPCR assays. Positive and negative extraction and PCR controls were appropriately positive and negative throughout.

All 100 samples from HIV-infected South African women were positive with the GAPDH qPCR (median *C*_t_ 24.3; range 20.7–29.8, thus all ≤30). None of the samples were positive with the generic haemoplasma haemominutum group qPCR assay but one sample (no. 153) was positive with the haemofelis group qPCR assay, with a *C*_t_ of 36.5 (this sample had a GAPDH *C*_t_ value of 24.4). Positive and negative extraction and PCR controls were appropriately positive and negative throughout. Sample number 153 underwent repeat generic haemoplasma haemofelis group qPCR twice more and was positive both times (*C*_t_ values of 39.1 and 37.7). Attempts were made to amplify and sequence the 16S rRNA and RNase P genes from the DNA extracted from sample number 153 using PCR as described previously (Peters *et al.*, 2008b; Tasker *et al.*, 2003c) but these were unsuccessful. Attempts were also made to concentrate the DNA from the remaining blood available by repeat extractions and elution into smaller volumes of buffer before reattempting amplification and sequencing, but again no sequencing data were generated.

### Blood smears

Swabs moistened in PBS to remove the blood smear material for subsequent DNA extraction gave acceptable results on qPCR (*n*=20); the *C*_t_ values for the feline haemoplasmas (*M. haemofelis* or ‘*Candidatus* M. haemominutum’), and feline 28S rDNA, obtained on fixed but unstained smears made with feline blood infected with these species were greater than those obtained on contemporaneous blood samples by 3.4–6.8 cycles (median 4.2). This median *C*_t_ difference of 4.2 roughly equates to an 18-fold difference in the amount of DNA detected on blood smears compared with blood samples, which is approximately that which would be expected if around 5 μl blood was used to make the blood smear compared with 100 μl used for DNA extraction from whole blood. Staining of the blood smears with Wright–Giemsa, Jenner–Giemsa, Giemsa or May Grünwald–Giemsa stains (*n*=6 for each stain), and subsequent DNA extraction and qPCR, resulted in *C*_t_ values for feline haemoplasma and 28S rDNA detection that differed from contemporaneous blood samples by 2.8–5.7 cycles (median 4.5), showing that the staining of the blood smears did not have a significant deleterious effect on either DNA extraction or subsequent qPCR. Additionally, spiking of the reference plasmid into the PCRs performed on DNA extracted from blood smears (*n*=6) did not provide any evidence of PCR inhibitors in these samples, with similar *C*_t_ values obtained for the reference plasmid whether smear DNA was present or not (data not shown).

All 15 blood smear samples that had been recruited from three previously reported human haemoplasma studies (Clark, 1975; Grétillat & Konarzewski, 1978; Yang *et al.*, 2000) gave negative results with the generic haemoplasma group assays, whilst haemoplasma-infected and uninfected blood smear extraction and PCR controls gave positive and negative results, respectively. All 15 blood smear samples gave positive results with the human GAPDH qPCR assay, but the *C*_t_ values ranged from 25.1 to 38.6 (median 29.2). Two of the 15 smear samples had human GAPDH *C*_t_ values of >33, which were considered unacceptable; both were from the Clark (1975) study (*C*_t_ values of 33.1 and 38.6).

## DISCUSSION

This report describes the development and application of generic qPCR assays, incorporating TaqMan probes, for the detection of haemoplasmas in human blood samples and blood smears. The high sensitivity and specificity of these assays make them ideal for screening large numbers of human samples. Of the 920 UK samples, 917 gave acceptable results (*C*_t_ ≤30) with the human GAPDH qPCR assay, but none of these were qPCR-positive for haemoplasmas. Since these samples were anonymized, we were unaware of what proportion of the samples were taken from anaemic or immunocompromised patients, which are thought to be at a higher risk of having haemoplasma infection (dos Santos *et al.*, 2008; Willi *et al.*, 2009). It is possible that the individuals sampled were not at high risk for haemoplasma infection.

Since *M. haemofelis*-like infection has been detected by PCR in the blood of an HIV-infected Brazilian man (dos Santos *et al.*, 2008), we sought to obtain blood samples from HIV-infected patients as we felt that such a group would comprise people at a higher risk of haemoplasma infection. Despite analysing 100 blood samples from South African women infected with HIV, only one sample gave a positive result with the generic haemofelis group qPCR. Unfortunately, the *C*_t_ value indicated a very low level of haemoplasma DNA in this sample and attempts to further characterize the haemoplasma species failed. Since all extraction and PCR negative controls were appropriately negative in the study, we believe that the positive result reflects infection with a haemoplasma species. Unfortunately, anonymization of the samples meant that we were unable to resample this patient.

The inclusion of the human GAPDH internal amplification control in this qPCR study enables us to be confident that amplifiable DNA was present in the majority of haemoplasma qPCR-negative samples; thus the negative haemoplasma qPCR results obtained are less likely to reflect problems with DNA extraction, PCR inhibition or operator error.

The development and optimization of a DNA extraction technique for the extraction of DNA from blood smears enabled us to analyse smears preserved from previous studies of suspected human haemoplasma infection (Clark, 1975; Grétillat & Konarzewski, 1978; Yang *et al.*, 2000). Despite acceptable amounts of GAPDH gene amplification from the majority (13/15) of these smears, no evidence of haemoplasma infection was detected by qPCR. Before the smears underwent DNA extraction, they were examined by light microscopy (S. T./H. N., data not shown) but no evidence of haemoplasma-like bodies was seen; when epierythrocyte-like structures were visible, these resembled stain precipitate or crenated edges of red blood cells. It is well known that cytology for the diagnosis of haemoplasma infection can have both poor specificity and sensitivity, and cytology is no longer recommended as a diagnostic method for haemoplasmosis due to its unreliability (Bauer *et al.*, 2008; Tasker *et al.*, 2003b; Westfall *et al.*, 2001). The results of this study support this, and strongly suggest that previous reports of human haemoplasmosis should be viewed with caution if the diagnosis was based on cytology alone without molecular confirmation. The smears in the current study that generated unacceptable levels of human GAPDH PCR product (*C*_t_ >33) could have yielded false-negative results with the haemoplasma qPCRs due to DNA extraction failure, damaged DNA, PCR inhibition or operator error. Indeed, DNA degradation was thought to be a possible problem when dealing with such old blood smears. It is encouraging, therefore, that acceptable amounts of human DNA were amplifiable from most of the blood smears, and it is of note that the two smears from which unacceptable amounts of human DNA were recovered were over 35 years old (Clark, 1975). Nevertheless, at least two smears from the same study (Clark, 1975) gave acceptable human GAPDH *C*_t_ values with concurrent negative haemoplasma qPCR results; since all of the smears from this study were derived from one patient, we believe that this patient showed no evidence of haemoplasmosis.

Our method for DNA extraction from blood smears offers a valuable tool for future PCR studies on suspected cases for which only blood smears are available. Optimization of the technique had to be based on the amplification of feline haemoplasmas and a feline 28S rDNA internal control, rather than human equivalents, due to the lack of haemoplasma-positive human samples. The median *C*_t_ difference between the blood smears and contemporaneous blood samples equated approximately to that expected from the different volumes of blood used for extraction. However, the precise volume of blood used to make blood smears will vary from smear to smear as will the number of nucleated cells present in the smears (from which the GAPDH gene is amplified). These variations are likely to contribute to the greater *C*_t_ differences identified for some samples during optimization.

These generic haemoplasma qPCR assays will be extremely useful for the detection of previously recognized haemoplasma species in samples, as well as the identification of novel haemoplasma species. A degree of cross-reactivity was detected between members of the haemominutum group and the haemofelis assay, although the *C*_t_ values obtained were later than with the group-specific assay. This cross-reactivity was not seen with all the samples from the haemominutum group that were used in the optimization and this is likely due to the number of haemoplasma copies present in the clinical samples that were available for testing. The cross-reactivity was not considered problematic as the intention of the study was to develop qPCRs that could be used to screen samples for any haemoplasma species. Indeed, very recently, both assays have been successfully used to identify novel haemoplasma species in rodents and primates, results for which will be presented elsewhere (S. T., personal communication). Since the assays produce only small PCR products, samples identified by this method require further characterization by amplification and sequencing of larger PCR products from the 16S rRNA gene and, preferably, an additional gene such as RNase P. However, as described in this report for sample number 153, further characterization may be limited by low levels of haemoplasma DNA. The inclusion of the human GAPDH gene qPCR helps validate negative results obtained in human samples. We found very little evidence of human haemoplasmosis in the samples screened in the current study, and previously reported cases of human haemoplasmosis based on cytology alone should be viewed with caution. However, recent reports of molecular evidence of human haemoplasma infection (Hu *et al.*, 2009; dos Santos *et al.*, 2008; Yang *et al.*, 2007), together with very recent findings from our laboratory using the described generic assay, strongly indicate that the search for haemoplasmosis in people should continue using reliable molecular techniques such as the qPCR assays described here, so that the significance of human haemotropic mycoplasma infections can be more accurately defined.

## Figures and Tables

**Table 1. t1:**
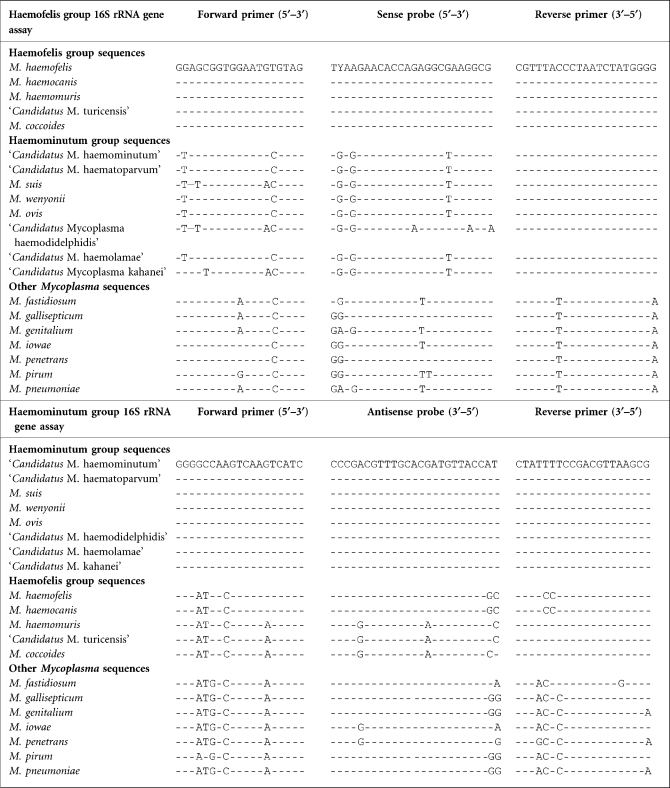
Oligonucleotide sequences of the primers and probes used for the detection of haemoplasma species to show mismatches in non-target haemoplasma and *Mycoplasma* species Dashes indicate identical bases with base differences indicated by the appropriate letter. The Y base in the probe for the haemofelis group assay indicated either a C or T base at this position. The orientation of the primers and probe is indicated for the appropriate sequences, i.e. 5′–3′ for the forward primer and sense probe and 3′–5′ for the reverse primer or antisense probe.

**Table 2. t2:** Details of primer and probe sequences used for the detection of haemoplasma species and human GAPDH

**Target**	**Forward primer**	**Reverse primer**	**5′ Fluorophore**	**Probe**	**3′ Quencher**	**Product size (bp)**	**% Reaction efficiency (sd)**	***R*^2^**
Haemofelis group 16S rRNA gene	GGAGCGGTGGAATGTGTAG	GGGGTATCTAATCCCATTTGC	FAM	TYAAGAACACCAGAGGCGAAGGCG	BHQ-1	114	100.5 (1.2)	0.99
Haemominutum group 16S rRNA gene	GGGGCCAAGTCAAGTCATC	GCGAATTGCAGCCTTTTATC	FAM	TACCATTGTAGCACGTTYGCAGCCC	BHQ-1	139	97.4 (1.9)	0.99
Human GAPDH gene	GAGTCAACGGATTTGGTCGT	AATGAAGGGGTCATTGATGG	Texas Red	CAGGGCTGCTTTTAACTCTGGCAAAGTGGA	BHQ-2	95	99.7 (0.4)	0.99

## Abbreviations

- *C*_t_, threshold cycle
- GAPDH, glyceraldehyde-3-phosphate dehydrogenase
- qPCR, quantitative real-time PCR

## References

[r1] Archer, G. L., Coleman, P. H., Cole, R. M., Duma, R. J. & Johnston, C. L., Jr (1979). Human infection from an unidentified erythrocyte-associated bacterium. N Engl J Med 301, 897–900. 113680 10.1056/NEJM197910253011701

[r2] Aubouy, A. & Carme, B. (2004). *Plasmodium* DNA contamination between blood smears during Giemsa staining and microscopic examination. J Infect Dis 190, 1335–1337. 15346346 10.1086/424529

[r3] Barker, E. N., Tasker, S., Day, M. J., Warman, S. M., Woolley, K., Birtles, R., Georges, K. C., Ezeokoli, C. D., Newaj-Fyzul, A. & other authors (2010). Development and use of real-time PCR to detect and quantify *Mycoplasma haemocanis* and “*Candidatus* Mycoplasma haematoparvum” in dogs. Vet Microbiol 140, 167–170. 19646827 10.1016/j.vetmic.2009.07.006 PMC2805721

[r4] Bauer, N., Balzer, H. J., Thure, S. & Moritz, A. (2008). Prevalence of feline haemotropic mycoplasmas in convenience samples of cats in Germany. J Feline Med Surg 10, 252–258. 18276180 10.1016/j.jfms.2007.12.004 PMC10832689

[r5] Bosnic, D., Baresic, M., Anic, B., Sentic, M., Cerovec, M., Mayer, M. & Cikes, N. (2010). Rare zoonosis (hemotrophic mycoplasma infection) in a newly diagnosed systemic lupus erythematosus patient followed by a *Nocardia asteroides* pneumonia. Braz J Infect Dis 14, 92–95. 20428663 10.1590/s1413-86702010000100019

[r6] Clark, K. G. (1975). A basophilic micro-organism infecting human red cells. Br J Haematol 29, 301–304. 1191550 10.1111/j.1365-2141.1975.tb01824.x

[r7] Dooley, J. R. (1980). Haemotropic bacteria in man. Lancet 2, 1237–1239. 10.1016/s0140-6736(80)92490-3 6108404

[r8] dos Santos, A. P., dos Santos, R. P., Biondo, A. W., Dora, J. M., Goldani, L. Z., de Oliveira, S. T., de Sa Guimaraes, A. M., Timenetsky, J., de Morais, H. A. & other authors (2008). Hemoplasma infection in HIV-positive patient, Brazil. Emerg Infect Dis 14, 1922–1924. 19046522 10.3201/eid1412.080964 PMC2634649

[r9] Duarte, M. I., Oliveira, M. S., Shikanai-Yasuda, M. A., Mariano, O. N., Takakura, C. F., Pagliari, C. & Corbett, C. E. (1992). *Haemobartonella*-like microorganism infection in AIDS patients: ultrastructural pathology. J Infect Dis 165, 976–977. 1569354

[r10] Grétillat, S. & Konarzewski, B. (1978). Presence of a prokaryote of the genus Haemobartonella Tyzzer and Weinman, 1939, in the blood of Nigerians in the Niamey region. Bull Soc Pathol Exot Filiales 71, 412–416 (in French ). 755536

[r11] Hoelzle, L. E. (2008). Haemotrophic mycoplasmas: recent advances in *Mycoplasma suis*. Vet Microbiol 130, 215–226. 18358641 10.1016/j.vetmic.2007.12.023

[r12] Hofmann-Lehmann, R., Meli, M. L., Dreher, U. M., Gonczi, E., Deplazes, P., Braun, U., Engels, M., Schupbach, J., Jorger, K. & other authors (2004). Concurrent infections with vector-borne pathogens associated with fatal hemolytic anemia in a cattle herd in Switzerland. J Clin Microbiol 42, 3775–3780. 15297529 10.1128/JCM.42.8.3775-3780.2004 PMC497630

[r13] Hu, Z., Yin, J., Shen, K., Kang, W. & Chen, Q. (2009). Outbreaks of hemotrophic mycoplasma infections in China. Emerg Infect Dis 15, 1139–1140. 19624945 10.3201/eid1507.090174 PMC2744233

[r14] Kallick, C. A., Levin, S., Reddi, K. T. & Landau, W. L. (1972). Systemic lupus erythematosus associated with *haemobartonella*-like organisms. Nat New Biol 236, 145–146. 4112548 10.1038/newbio236145a0

[r15] Kallick, C. A., Thadhani, K. C. & Rice, T. W. (1980). Identification of Anaplasmataceae (Haemobartonella) antigen and antibodies in systemic lupus erythematosus. Arthritis Rheum 23, 197–205. 6153897 10.1002/art.1780230210

[r16] Neimark, H., Johansson, K. E., Rikihisa, Y. & Tully, J. G. (2001). Proposal to transfer some members of the genera *Haemobartonella* and *Eperythrozoon* to the genus *Mycoplasma* with descriptions of ‘*Candidatus* Mycoplasma haemofelis’, ‘*Candidatus* Mycoplasma haemomuris’, ‘*Candidatus* Mycoplasma haemosuis’ and ‘*Candidatus* Mycoplasma wenyonii’. Int J Syst Evol Microbiol 51, 891–899. 11411711 10.1099/00207713-51-3-891

[r17] Neimark, H., Barnaud, A., Gounon, P., Michel, J. C. & Contamin, H. (2002). The putative haemobartonella that influences *Plasmodium falciparum* parasitaemia in squirrel monkeys is a haemotrophic mycoplasma. Microbes Infect 4, 693–698. 12067828 10.1016/s1286-4579(02)01588-5

[r18] Neimark, H., Hoff, B. & Ganter, M. (2004). *Mycoplasma ovis* comb. nov. (formerly *Eperythrozoon ovis*), an epierythrocytic agent of haemolytic anaemia in sheep and goats. Int J Syst Evol Microbiol 54, 365–371. 15023944 10.1099/ijs.0.02858-0

[r19] Peters, I. R., Helps, C. R., Willi, B., Hofmann-Lehmann, R. & Tasker, S. (2008a). The prevalence of three species of feline haemoplasmas in samples submitted to a diagnostics service as determined by three novel real-time duplex PCR assays. Vet Microbiol 126, 142–150. 17689890 10.1016/j.vetmic.2007.06.017

[r20] Peters, I. R., Helps, C. R., McAuliffe, L., Neimark, H., Lappin, M. R., Gruffydd-Jones, T. J., Day, M. J., Hoelzle, L. E., Willi, B. & other authors (2008b). RNase P RNA gene (*rnpB*) phylogeny of hemoplasmas and other *Mycoplasma* species. J Clin Microbiol 46, 1873–1877. 18337389 10.1128/JCM.01859-07 PMC2395117

[r21] Pretorius, A. M., Sommer, A. P., Aho, K. M. & Kajander, E. O. (2004). HIV and nanobacteria. HIV Med 5, 391–393. 15544689 10.1111/j.1468-1293.2004.00242.x

[r22] Puntaric, V., Borcic, D., Vukelic, D., Jeren, T., Burek, V., Wikerhauser, T. & Richter, B. (1986). Eperythrozoonosis in man. Lancet 2, 868–869. 10.1016/s0140-6736(86)92910-7 2876318

[r23] Puntaric, V., Borcic, D., Bejuk, D., Vrhovec, B., Madic, J., Busch, K. & Richter, B. (1994). Haemotropic bacteria in man. Lancet 343, 359–360. 10.1016/s0140-6736(94)91202-5 7905172

[r24] Stoffregen, W. C., Alt, D. P., Palmer, M. V., Olsen, S. C., Waters, W. R. & Stasko, J. A. (2006). Identification of a haemomycoplasma species in anemic reindeer (*Rangifer tarandus*). J Wildl Dis 42, 249–258. 16870847 10.7589/0090-3558-42.2.249

[r25] Sykes, J. E., Owens, S. D., Terry, J. C., Lindsay, L. L. & Pusterla, N. (2008). Use of dried blood smears for detection of feline hemoplasmas using real-time polymerase chain reaction. J Vet Diagn Invest 20, 616–620. 18776095 10.1177/104063870802000513

[r26] Tasker, S. (2010). Haemotropic mycoplasmas: what's the real significance in cats? J Feline Med Surg 12, 369–381. 20417898 10.1016/j.jfms.2010.03.011 PMC2880789

[r27] Tasker, S., Helps, C. R., Day, M. J., Gruffydd-Jones, T. J. & Harbour, D. A. (2003a). Use of real-time PCR to detect and quantify *Mycoplasma haemofelis* and “*Candidatus* Mycoplasma haemominutum” DNA. J Clin Microbiol 41, 439–441. 12517888 10.1128/JCM.41.1.439-441.2003 PMC149582

[r28] Tasker, S., Binns, S. H., Day, M. J., Gruffydd-Jones, T. J., Harbour, D. A., Helps, C. R., Jensen, W. A., Olver, C. S. & Lappin, M. R. (2003b). Use of a PCR assay to assess prevalence and risk factors for *Mycoplasma haemofelis* and ‘*Candidatus* Mycoplasma haemominutum’ in cats in the United Kingdom. Vet Rec 152, 193–198. 12620033 10.1136/vr.152.7.193

[r29] Tasker, S., Helps, C. R., Day, M. J., Harbour, D. A., Shaw, S. E., Harrus, S., Baneth, G., Lobetti, R. G., Malik, R. & other authors (2003c). Phylogenetic analysis of hemoplasma species – an international study. J Clin Microbiol 41, 3877–3880. 12904408 10.1128/JCM.41.8.3877-3880.2003 PMC179806

[r30] Tasker, S., Caney, S. M. A., Day, M. J., Dean, R. S., Helps, C. R., Knowles, T. G., Lait, P. J. P., Pinches, M. D. G. & Gruffydd-Jones, T. J. (2006a). Effect of chronic FIV infection, and efficacy of marbofloxacin treatment, on *Mycoplasma haemofelis* infection. Vet Microbiol 117, 169–179. 16876338 10.1016/j.vetmic.2006.06.015

[r31] Tasker, S., Caney, S. M. A., Day, M. J., Dean, R. S., Helps, C. R., Knowles, T. G., Lait, P. J. P., Pinches, M. D. G. & Gruffydd-Jones, T. J. (2006b). Effect of chronic FIV infection, and efficacy of marbofloxacin treatment, on ‘*Candidatus* Mycoplasma haemominutum’ infection. Microbes Infect 8, 653–661. 16483821 10.1016/j.micinf.2005.08.015

[r32] Tasker, S., Peters, I. R., Day, M. J., Willi, B., Hofmann-Lehmann, R., Gruffydd-Jones, T. J. & Helps, C. R. (2009). Distribution of feline haemoplasmas in blood and tissue following experimental infection. Microb Pathog 47, 334–340. 19782126 10.1016/j.micpath.2009.09.009 PMC2791849

[r33] Untergasser, A., Nijveen, H., Rao, X., Bisseling, T., Geurts, R. & Leunissen, J. A. M. (2007). Primer3Plus, an enhanced web interface to Primer3. Nucleic Acids Res 35, W71–W74. 17485472 10.1093/nar/gkm306 PMC1933133

[r34] Westfall, D. S., Jensen, W. A., Reagan, W. J., Radecki, S. V. & Lappin, M. R. (2001). Inoculation of two genotypes of *Haemobartonella felis* (California and Ohio variants) to induce infection in cats and the response to treatment with azithromycin. Am J Vet Res 62, 687–691. 11341386 10.2460/ajvr.2001.62.687

[r35] Willi, B., Filoni, C., Catao-Dias, J. L., Cattori, V., Meli, M. L., Vargas, A., Martinez, F., Roelke, M. E., Ryser-Degiorgis, M. P. & other authors (2007). Worldwide occurrence of feline hemoplasma infections in wild felid species. J Clin Microbiol 45, 1159–1166. 17301277 10.1128/JCM.02005-06 PMC1865832

[r36] Willi, B., Meli, M. L., Luthy, R., Honegger, H., Wengi, N., Hoelzle, L. E., Reusch, C. E., Lutz, H. & Hofmann-Lehmann, R. (2009). Development and application of a universal hemoplasma screening assay based on the SYBR Green PCR principle. J Clin Microbiol 47, 4049–4054. 19828748 10.1128/JCM.01478-09 PMC2786680

[r37] Yang, D., Tai, X., Qiu, Y. & Yun, S. (2000). Prevalence of *Eperythrozoon* spp. infection and congenital eperythrozoonosis in humans in Inner Mongolia, China. Epidemiol Infect 125, 421–426. 11117967 10.1017/s0950268899004392 PMC2869616

[r38] Yang, Z., Yuan, C., Yu, F. & Hua, X. (2007). Haemotrophic mycoplasma: review of aetiology and prevalence. Rev Med Microbiol 18, 1–3.

[r39] Yuan, C., Liang, A., Yu, F., Yang, Z., Li, Z., Zhu, J., Cui, L., Han, Y. & Hua, X. (2007). *Eperythrozoon* infection identified in an unknown aetiology anaemia patient. Ann Microbiol 57, 467–469.

[r40] Yuan, C. L., Liang, A. B., Yao, C. B., Yang, Z. B., Zhu, J. G., Cui, L., Yu, F., Zhu, N. Y., Yang, X. W. & other authors (2009). Prevalence of *Mycoplasma suis* (*Eperythrozoon suis*) infection in swine and swine-farm workers in Shanghai, China. Am J Vet Res 70, 890–894. 19566474 10.2460/ajvr.70.7.890

